# The effects of process parameters on polydopamine coatings employed in tissue engineering applications

**DOI:** 10.3389/fbioe.2022.1005413

**Published:** 2022-09-12

**Authors:** Soulmaz Sarkari, Mehran Khajehmohammadi, Niyousha Davari, Dejian Li, Baoqing Yu

**Affiliations:** ^1^ Department of Biomedical Engineering, Science and Research Branch, Islamic Azad University, Tehran, Iran; ^2^ Department of Mechanical Engineering, Faculty of Engineering, Yazd University, Yazd, Iran; ^3^ Medical Nanotechnology and Tissue Engineering Research Center, Yazd Reproductive Sciences Institute, Shahid Sadoughi University of Medical Sciences, Yazd, Iran; ^4^ Department of Life Science Engineering, Faculty of New Sciences and Technologies, University of Tehran, Tehran, Iran; ^5^ Department of Orthopedics, Shanghai Pudong Hospital, Fudan University Pudong Medical Center, Shanghai, China; ^6^ Department of Orthopedics, Shanghai Pudong New Area People’s Hospital, Shanghai, China

**Keywords:** polydopamine, surface modification, process parameters, tissue engineering, biomaterial, coating

## Abstract

The biomaterials’ success within the tissue engineering field is hinged on the capability to regulate tissue and cell responses, comprising cellular adhesion, as well as repair and immune processes’ induction. In an attempt to enhance and fulfill these biomaterials’ functions, scholars have been inspired by nature; in this regard, surface modification via coating the biomaterials with polydopamine is one of the most successful inspirations endowing the biomaterials with surface adhesive properties. By employing this approach, favorable results have been achieved in various tissue engineering-related experiments, a significant one of which is the more rapid cellular growth observed on the polydopamine-coated substrates compared to the untreated ones; nonetheless, some considerations regarding polydopamine-coated surfaces should be taken into account to control the ultimate outcomes. In this mini-review, the importance of coatings in the tissue engineering field, the different types of surfaces requiring coatings, the significance of polydopamine coatings, critical factors affecting the result of the coating procedure, and recent investigations concerning applications of polydopamine-coated biomaterials in tissue engineering are thoroughly discussed.

## 1 Introduction

Material selection and other critical qualities of scaffolds, including possessing an adhesive surface, biodegradability, biocompatibility, mechanical stability, etc., must be meticulously considered so that the various tissues can be successfully regenerated (Zhang et al., 2019; Ghorbani et al., 2020b). Concerning this subject, surface engineering of biomaterials using various techniques is gaining increasing prominence in tissue engineering (TE) applications, and surface modification, as a popular surface engineering strategy, is utilized to alter the microenvironment of cells and transform the scaffolds’ surface into an adhesive one (Arab-Ahmadi et al., 2021; Davari et al., 2022).

Compared to other coating substances, including proteins (Gennadios, 2002), particular peptide sequences (Pagel and Beck-Sickinger, 2017), ceramics (Montazerian et al., 2022), and metals (Yin et al., 2021), polydopamine (PDA), a melanin-like mussel-inspired coating polymer (Ghorbani et al., 2022), possesses a variety of desirable properties, such as outstanding adhesion features (Ghalandari et al., 2021; Feinberg and Hanks, 2022), extraordinary hydrophilicity (Li et al., 2018), biodegradability (Zhang and King, 2020), uniform shape (Saeed et al., 2021), biocompatibility (Zeng et al., 2020), and thermal stability (Yim et al., 2022). Furthermore, its biological properties, including enhanced cellular proliferation, improved bioactivity, free-radical scavenging activities, metal ion chelating capacity, and anti-bacterial capability, originate from its catecholamine and hydroxyl functional groups (Yu et al., 2017; Ghorbani et al., 2019b, 2019a; Deng et al., 2021). Notably, solvent selection is of paramount importance for solution-based chemical reactions associated with polymers like PDA. Water and ethanol have been broadly employed among various solvents (Jiang et al., 2014; Nieto et al., 2021). A self-assembled PDA layer can be formed on the surfaces of a variety of materials, like metals, ceramics, polymers, oxides, and semiconductors, when soaked in a PDA’s weakly alkaline aqueous solution (10 mM Tris-HCl, pH∼8.5) without the addition of sophisticated connecting agents (Kao et al., 2018; Yang et al., 2019; Lee et al., 2020; Li et al., 2021b; Park et al., 2021). Nonetheless, the volume ratio of water to alcohol strongly influences the PDA spheres’ synthesis. It was illustrated that neither did PDA microspheres form in the pure ethanol nor had a perfect shape in the pure water; in essence, when ethanol and water were mixed with a ratio of 30:70, formation happened in the best way possible (Yan et al., 2013). Insufficient conductivity has ever been the major weakness of PDA (Mei et al., 2020). Since the pros of PDA outweigh its cons, various groups of scientists have modified the biomaterials’ surfaces by employing PDA with the aim of augmenting their surface performance. Therefore, within the last couple of years, PDA has been in the spotlight, with diverse applications in the field of biomedical engineering (Lynge et al., 2011), including drug delivery (Huang et al., 2018), implants (Jia et al., 2019), surface engineering (Yang et al., 2015), cancer therapy (Abdollahi et al., 2022; Honmane et al., 2022), TE (bone (Huang et al., 2016; Kaushik et al., 2020), cartilage (Huang et al., 2021), muscle (Zhou et al., 2021), skin (Yazdi et al., 2022), tendon (Lin et al., 2019), and neuron (Qian et al., 2018; Yan et al., 2020)), and microfluidic systems (Niculescu et al., 2021).

The process parameters of PDA considerably impact the coating’s quality and characteristics; thus, the experimental factors, such as coating time, temperature, pH, and DA’s initial concentration, are properly adjusted so that a coating layer with outstanding features can be formed (Ghorbani et al., 2020c).

Within this paper, firstly, the coatings’ significance in the TE field and various kinds of surfaces in need of coatings are comprehensively reviewed. Afterward, the PDA coatings’ importance is exhaustively discussed. Ultimately, the parameters that are regulated to improve the properties of coatings and the employment of these interesting coatings in TE by introducing relevant case studies are explained.

## 2 Polydopamine-coated surfaces

Synthetic polymers, such as polylactic-co-glycolic acid (PLGA) (Ghorbani et al., 2020c), polyurethane (PU) (Merceron et al., 2015), polycaprolactone (PCL) (Perez-Puyana et al., 2021), polydimethylsiloxane (PDMS) (Cunniffe et al., 2019), poly-l-lactic acid (PLA) (Jenkins and Little, 2019), polyvinyl alcohol (PVA) (Rodríguez-Rodríguez et al., 2020), polyhydroxyalkanoates (PHAs) (Aguilar-Rabiela et al., 2021; Gregory et al., 2022), and polyamide (Razaviye et al., 2022) are frequently utilized in the fabrication of different scaffolds due to their superior characteristics, comprising customizable degradation rate, excellent processability, great mechanical properties, and wide *in-vitro* and *in-vivo* availability. Nevertheless, synthetic polymers typically have poor cell adhesion properties; consequently, combinations with natural polymers or various modifications are common approaches to tackle this obstacle (Terzopoulou et al., 2022). Besides, these modifications are also applied to natural polymers, such as chitosan (Bock et al., 2020), cellulose (Nishida and Koga, 2018), peptides (Ji and Parquette, 2020), etc., to enhance their features.

Boosting biomaterials’ performance, modifying their physicochemical properties, and broadening the applications of them are possible by conducting the surface modification procedure (Salatin et al., 2015). Considerable effort has been dedicated to the substances’ surface modification via employing various techniques, such as self-assembly monolayer (Kehr et al., 2015), plasma surface modification (Ghorbani et al., 2020d), and layer-by-layer assembly (Liu et al., 2016). Despite these approaches’ multiple benefits, they suffer from some drawbacks: limited chemical characterization, restricted shape and dimension of the resultant substances, complicated equipment, and complex synthesis processes (Liu et al., 2016). As an alternative, a coating strategy based upon PDA, formed through covalent polymerization and non-covalent self-assembly (Hong et al., 2012), is widely employed.

The convoluted structure of PDA incorporates a mixture of diverse oligomers formed as a result of dopamine (DA) oxidation, including indole units with varying degrees of oxidation, carboxylic acid and amino groups, imine units, indolic/catecholic π-systems, quinone residues, phenol groups, and open-chain DA molecules (Liu et al., 2014; Ghorbani et al., 2019c). Of note, the functional groups in the PDA structure (catechol, imine, and amine) are employed to reinforce the interaction between the biomaterial and the tissue. What’s more, within its irregular, cross-linked network with mixed bonding configurations, radicals are located at quinone residues (Du et al., 2017). Owing to the presence of a series of constant π-electron free radicals, PDA is paramagnetic and operates analogously to a radical trap (Kim et al., 2021b).

As a transitional surface, PDA enables the adhesion of other materials to scaffolds without demanding pre-functionalization. This phenomenon is mainly because of the quinone functional groups that are: 1) electron-deficient and 2) capable of participating in Schiff base reactions occurring on amines and stimulating the Michael addition reactions with nucleophilic groups, including amines and thiols (Rizzo and Kehr, 2021). Notably, the PDA surfaces’ sensitivity towards the nucleophiles relies on the catechol/quinone equilibrium; therefore, the generated alkaline pH speeds up conjugation reactions (Batul et al., 2017). Other functional groups within the structure or generated by PDA serve pivotal roles; for instance, amine groups are involved in displacing hydrated minerals on the surface as well as bringing catechols closer to the surface to create strong molecular interactions (Lee et al., 2019). Another example is the hydroxyl and amine groups produced by PDA on the scaffolds that can promptly release signals to promote integrin binding, resulting in the increment of osteogenic activities (Ghorbani et al., 2020c). The more binding functional groups on the surface, the higher probability of the molecules and inorganic ions loading, leading to the improvement of cellular attachment (Richbourg et al., 2019).

## 3 Polydopamine coating’s key process parameters in tissue engineering applications

Brimming with outstanding features, PDA coatings have been extensively employed in TE-related investigations. In this regard, several experiments have achieved favorable outcomes when utilizing this coating compared with the others (Sharma et al., 2019; Kopeć et al., 2022). Within a novel study, scaffolds were coated with PDA and gelatin to evaluate the anti-corrosion property. The PDA-coated and gelatin-coated samples were biocompatible and displayed corrosion current density (I_corr_) of 2.95 × 10^–3^ and 3.72 × 10^–3^ $\frac{\text{A}}{{\text{cm}}^{2}}$ , respectively, indicating that the PDA coating layer could delay the corrosive process (Zhou et al., 2019).

**FIGURE 1:**
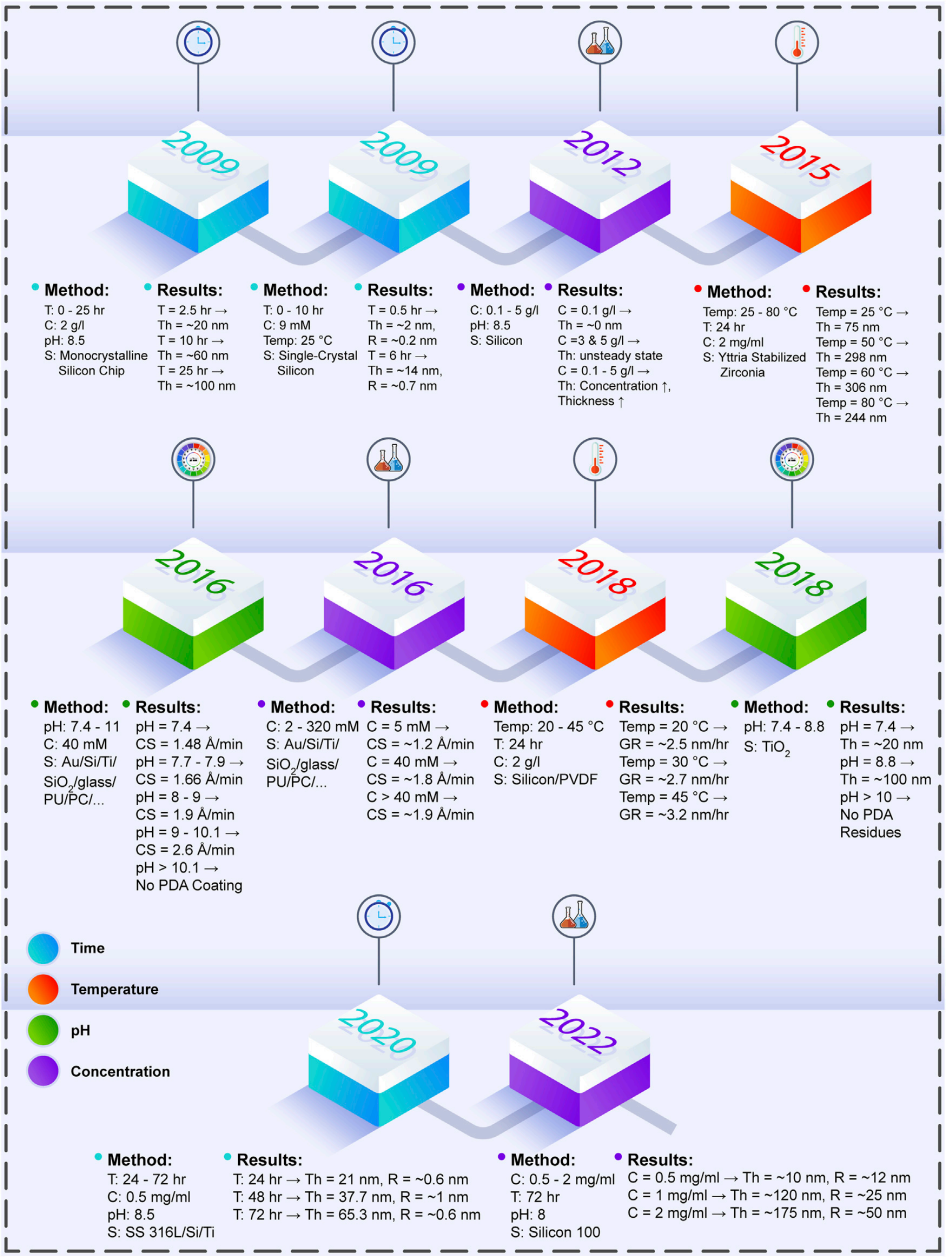
The effects of PDA process parameters on the produced PDA layer, which are investigated in various experiments throughout the years (T: time, C: concentration, S: surface, Th: thickness, Temp: temperature, R: roughness, Au: aurum, Ti: titanium, Si: silicon, PU: polyurethane, PC: polycarbonate, CS: coating speed, PVDF: polyvinylidene fluoride, GR: coating layer growth rate, SS 316L: stainless steel 316L) (Li et al., 2009; Ou et al., 2009; Ball et al., 2012; Zain et al., 2015; Hong et al., 2016; Habibi Rad et al., 2018; Hong et al., 2018; Zhou et al., 2020; Szewczyk et al., 2022).

For PDA polymerization on the surface of the biomaterials, multiple factors should be considered to achieve the desired outcomes in research cases. Among these parameters, coating time, temperature, pH of Tris-HCl, and initial DA concentration in the DA/Tris-HCl buffer solution are discussed in the following. Table 1 presents these determining factors and their impacts upon the formed PDA layer. Furthermore, Figure 1 illustrates the outline briefly describing the case studies in terms of the process parameters and their effects.

**TABLE 1:** PDA coating parameters and their effects on the formed PDA layer.

PDA coating parameters		Thickness increment	Thickness reduction	Normal value	Polymerization rate increase	Roughness increment	Refs
Coating time		✓	-	24 h	-	✓	Li et al. (2009); Ou et al. (2009); Dang et al. (2015); Ghorbani et al. (2020a); Liu et al. (2020); Kim et al. (2021a); Felfel et al. (2021); Zia et al. (2021)
Temperature		✓	-	Ambient temperature	✓	✓	Ou et al. (2009); Jiang et al. (2011); Proks et al. (2013); Zhou et al. (2014); Schaubroeck et al. (2015); Zain et al. (2015); Deng et al. (2018); Habibi Rad et al. (2018); Malollari et al. (2019); Oymaci et al. (2020); Davidsen et al. (2021)
pH of Tris-HCl	**alkaline**	✓	-	8.5	✓	-	Yang et al. (2014); Hong et al. (2016); Dong et al. (2018); Hong et al. (2018); Salomäki et al. (2018); Mei et al. (2021); Gan et al. (2022); Szewczyk et al. (2022)
	**acidic**	-	✓	-	-	-	
DA initial concentration		✓	-	2 $\frac{\text{mg}}{\text{ml}}$	✓	✓	Ball et al. (2012); Ding et al. (2014); Hong et al. (2016); Dong et al. (2018); Oymaci et al. (2020); Wu et al. (2020); Li et al. (2021a); Wang et al. (2022)

### 3.1 Coating time

**FIGURE 2:**
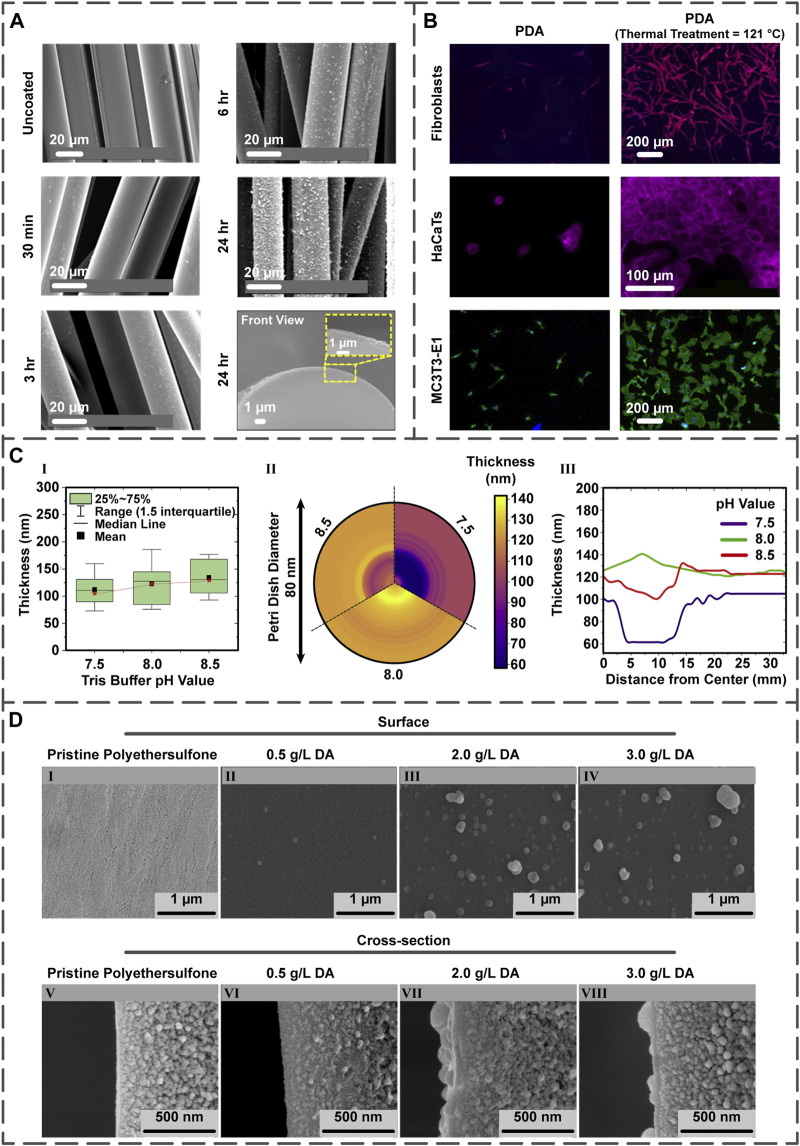
**(A)** The influence of PDA coating time upon roughness and thickness of the PDA layer on the PGF (reproduced content is open access) (Felfel et al., 2021). **(B)** Post-treatment of the PDA layer present on the PDA-coated Ti samples and the quantification of fibroblast, HaCaT, and MC3T3-E1 cells on the surfaces (reproduced content is open access) (Davidsen et al., 2021). **(C)** The different pH values of Tris buffer and their effects on the film thickness illustrated in the comparative chart **(I)**, mapping chart **(II)**, and line chart **(III)** (reproduced content is open access) (Szewczyk et al., 2022). **(D)** The DA concentration impact on the morphology of coating displayed by field emission scanning electron microscopy images. Surface and cross-section images of (**I**,**V**) non-coated polyethersulfone membranes as well as membranes coated with PDA possessing DA concentrations of (**II**,**VI**) 0.5 $\frac{\text{g}}{\text{L}}$ , (**III**,**VII**) 2.0 $\frac{\text{g}}{\text{L}}$ and (**IV**,**VIII**) 3.0 $\frac{\text{g}}{\text{L}}$ at 10^5^ X and 25 × 10^4^ X magnifications, respectively (reproduced content is open access) (Oymaci et al., 2020).

The PDA coating process commonly occurs at room temperature in pH 8.5 for 24 h with the initial concentration of 2 $\frac{\text{mg}}{\text{ml}}$ ; in this regard, an investigation on the effects of PDA coating upon PVA/PU-polyaniline matrix is an interesting example in which the matrix was immersed into the DA solution under normal mentioned conditions. Scholars reported that the coating enhanced mechanical characteristics; more specifically, the resultant scaffold’s tensile strength was 34.06 ± 1 MPa compared to the 29.51 ± 1.63 MPa strength shown without coating. Besides, the surface modification with PDA significantly improved the osseointegration and promoted the adhesion and differentiation of rat bone marrow mesenchymal stem cells (BMSCs), all of which were beneficial for bone TE (Ghorbani et al., 2020a). Nonetheless, the coating time is one of the main parameters influencing the produced PDA layer; in essence, this factor exerts a direct impact on the thickness of PDA. The more the coating time, the more PDA thickness (Li et al., 2009). As an instance, Ou and co-workers (Ou et al., 2009) coated modified silicon (Si) substrates with PDA and concluded that with the prolonging of deposition time, the coating got thicker, and the I_corr_ decreased from 247.73 $\frac{\text{A}}{{\text{cm}}^{2}}$ in 2 h to 11.5.45 $\frac{\text{A}}{{\text{cm}}^{2}}$ in 10 h. The impact of coating time was analyzed in a research project to make phosphate glass fiber (PGF) suitable for the PCL/PGF composites formation. Firstly, PGF was dipped in the DA hydrochloride aqueous solution for varying periods (30 min–24 h), followed by the formation of PCL/PGF scaffolds utilizing the *in-situ* polymerization method. With increasing the coating time, the PDA quantity on the fiber surface enhanced; thus, when the time was set to 24 h, the PDA thickness augmented to 46 nm in comparison with 18 nm after 6 h. Furthermore, it was concluded that the combination of annealing and coating processes could yield better results for the bone TE applications compared to the sole coating procedure. Regarding this matter, flexural strength and modulus of annealed/coated, annealed/non-coated, and non-annealed fibers retained at nearly 70–75%, less than 20%, and less than 10% of the day one characteristics (strength: 120 MPa and modulus: 14 GPa) after 56 days, respectively. The achieved features associated with annealed-coated fibers successfully met the target for bone repair for the upper extremities at least (Figure 2A) (Felfel et al., 2021).

Multiple studies have suggested the direct influence of coating time on the PDA layer roughness (Ou et al., 2009; Dang et al., 2015; Liu et al., 2020). Concerning this matter, Kim and co-workers engineered high robust PDA-coated polybenzimidazole membranes under different coating times (5, 10, and 30 min). The atomic force microscope image of the PDA layer illustrated that with increasing time, the roughness enhanced from nearly 1 nm in 5 min to 4 nm in 30 min (Kim et al., 2021a).

As an intermediate layer, PDA coating has been employed in various experiments. Recently, Zia et al. (Zia et al., 2021) used a PDA layer between chitosan and porous PLA via applying different polymerization times (1, 6, 12, 18, and 24 h). It was observed that the PDA layer thickness increased with time; to delineate, although some uncovered areas lacking the PDA layer were found after 1 h, a layer with an acceptable thickness appeared when polymerization continued for 6 h. Moreover, the scaffold’s diameter and hydrophilicity were both enhanced by time; to elucidate on, the diameter increased from 1.2 μm after 6 h to 1.8 μm after 24 h, and the water contact angle decreased from 85° at 6 h to 70.6° at 24 h.

### 3.2 Temperature

The process temperature has been shown to improve thickness, roughness, as well as polymerization rate and shorten the polymerization time of the PDA layer. In an investigation conducted by a group of researchers (Zhou et al., 2014), it was illustrated that among various temperatures (25, 37, and 60°C), 60°C and 300 $\frac{\text{r}}{\text{min}}$ stirring could hasten the time of polymerization (30 min) without triggering chemical changes in the PDA layer compared to the 24-h normal approach. Regarding the comparison between dynamic and static conditions, PDA-coated surfaces prepared under the dynamic condition (stirring) in the range of 1–8 h displayed some superior features like higher thickness and lower contact angle compared to the ones fabricated under the static condition during 24 h. Furthermore, PDA coating endowed the scaffolds with favorable cytocompatibility since cultivated MG-63 osteoblast cells were highly viable.

Another effect of temperature is the increment of the PDA layer’s thickness (Zain et al., 2015; Oymaci et al., 2020) and roughness (Schaubroeck et al., 2015; Oymaci et al., 2020), which was carefully assessed in a project. It was reported that the coating thickness on several materials, such as Si/polyvinylidene fluoride (PVDF), was augmented by raising the temperature; to be more precise, thickness increased from nearly 20 nm at 20°C to 65 nm at 45°C. It is also noteworthy to mention that the modified surfaces’ water contact angle decreased with augmenting the reaction temperature (from 63.9° at 20°C to 53.2° at 45°C). Regarding the roughness, further tests indicated that the layer roughness increased from 6.4 ± 0.9 nm (20°C) to 10.4 ± 1.6 nm (45°C) (Jiang et al., 2011).

Concerning the influence of temperature on the polymerization rate, several studies have reported the rate enhancement with temperature raising (Deng et al., 2018; Habibi Rad et al., 2018). In a novel experiment in which PDA-coated Si surfaces were fabricated, the mentioned effect was investigated within a temperature range of 25–35°C. They found that higher temperature led to the accelerated rate; indeed, polymerization speed increased from 1.8 $\frac{\text{nm}}{\text{hr}}$ at 25°C to 2.2 $\frac{\text{nm}}{\text{hr}}$ at 35°C (Ou et al., 2009).

Improving the coating stability can be achieved by the coating’s thermal treatment. In essence, the thermal treatment affects the number of quinone and amine groups at the PDA surface (Proks et al., 2013; Malollari et al., 2019). Within an intriguing study, titanium (Ti)/Si wafer samples were coated via PDA; specifically, some were thermally treated by storing at 121°C for 24 h, while others were post-treated by 2-week storing at room temperature. In both treatments, the numbers of the quinone and primary amine groups increased and decreased, respectively, improving layer stability and favoring cellular proliferation. The proliferation of fibroblast, human keratinocytes (HaCaT), and MC3T3-E1 cells on post-treated PDA-coated samples was more enhanced in comparison with the PDA-coated ones (Figure 2B) (Davidsen et al., 2021).

### 3.3 pH of Tris-HCl

In the synthesis process of PDA coating, various experiments have reported the creation of a weak alkaline environment (pH > 8.5) with oxygen as the oxidant in the Tris-HCl buffer presence. When oxygen is constantly present as an oxidant in the reaction, an even coating of PDA is formed. As another synthesis method, creating an oxygen gradient can lead to the formation of a PDA gradient coating (Lee et al., 2019). In some synthesis approaches with other oxidants, the pH may vary between weakly acidic and neutral (Wei et al., 2010; Bernsmann et al., 2011; Ghorbani et al., 2021). Furthermore, PDA can be formed via self-polymerization within an acidic environment employing hydrothermal methods (160°C in 6 h) (Zheng et al., 2015).

The pH value directly affects the PDA layer thickness (Hong et al., 2018) since the initial DA oxidation depends on the amount of pH (Yang et al., 2014; Salomäki et al., 2018). Regarding this subject, the influence of pH on the PDA two-dimensional films’ thickness was examined by choosing pH values of 7.5, 8.0, and 8.5. Interestingly, the lowest thickness (24 nm) was achieved at pH 7.5, whereas the highest one (35 nm) was obtained at pH 8.5. However, the most favorable result was found in pH 8.0 because of the fewer variations between the central and external regions of the film (Figure 2C) (Szewczyk et al., 2022).

Concerning the impact of pH on the polymerization rate, multiple investigations have revealed that the rate increases with the pH increment (Dong et al., 2018; Mei et al., 2021). Within an innovative project, sprayable PDA with ultra-fast polymerization was employed to coat solid surfaces. In this regard, the pH was selected in the range of 7–11, and when it went beyond 10.1, no PDA layer could be formed. Nevertheless, the polymerization speed enhanced from 1.48 $\frac{\text{A}{}^{\circ}}{\text{min}}$ at pH 7.4 to 2.6 $\frac{\text{A}{}^{\circ}}{\text{min}}$ at pH 9–10.1 (Hong et al., 2016).

Given that better outcomes concerning the PDA polymerization have been produced in the alkaline environment, most studies have concentrated on this pH range. For example, as a modification factor, PDA was grafted onto the hyaluronic acid (HA) chains under a pH value of 8.0, followed by the production of hydrogel scaffolds through incorporating PDA/HA complex in a dual cross-linked collagen type I (Col I) matrix for use in cartilage regeneration. The final results showed that the employed PDA coating enhanced BMSCs’ proliferation, improved cellular affinity, and induced chondrogenic differentiation of cells compared to those of the cell-seeded scaffolds without the PDA layer. Additionally, it augmented anti-inflammation capacity, immune modulation ability, and *in-vivo* cartilage repair in the rabbit model (Gan et al., 2022).

### 3.4 Concentration

Like other discussed parameters, concentration is another critical factor impacting the final PDA layer. Of note, the concentration of the DA monomer must be at least 2 $\frac{\text{mg}}{\text{ml}}$ to achieve a layer of PDA. In a novel project, magnesium discs were coated with a calcium-deficient HA (CDHA) layer using hydrothermal treatment. Afterward, vascular endothelial growth factor was bound on the CDHA surface through PDA’s covalent bonds (2 $\frac{\text{mg}}{\text{ml}}$ and pH 8.5). It was concluded that the surface morphology altered when coating with the PDA layer was smoother, and the coating decreased the rate of magnesium degradation; therefore, a desirable environment was created for the growth factor, indicating the good biocompatibility of this scaffold for prolonged usage (Li et al., 2021a).

For investigating the effect of concentration on the PDA layer, the porous Col I-HA scaffold was modified via immersing into various DA concentrations (0.5, 1.0, and 2.0 $\frac{\text{mg}}{\text{ml}}$ ) prepared in 10 mM Tris-HCl buffer solution at ambient temperature. All coated sponges maintained their porous structure, and the scaffold with 2.0 $\frac{\text{mg}}{\text{ml}}$ DA concentration had the highest swelling rate, largest reactive oxygen species scavenging capacity, excellent antioxidant property resulting from the PDA’s catechol groups, good procoagulant effect, and higher promotion of diabetic wound healing in the rat model compared to other groups (Wang et al., 2022).

With respect to the effect of concentration on the polymerization rate, the rate boosts with the concentration increment (Hong et al., 2016). For instance, in the DA concentration range of 0.1–5 $\frac{\text{g}}{\text{L}}$ , the highest polymerization speed, 4 $\frac{\text{nm}}{\text{hr}}$ , was obtained in the concentration of 5 $\frac{\text{g}}{\text{L}}$ (Ball et al., 2012).

The thickness and roughness of the PDA layer can be controlled by tuning this factor; regarding this matter, several studies have stated that lower concentrations result in lower PDA’s thickness (Dong et al., 2018; Wu et al., 2020) and roughness (Ball et al., 2012; Ding et al., 2014). To evaluate these phenomena, a team of scientists (Oymaci et al., 2020) coated the polyethersulfone membranes with PDA possessing diverse concentrations (0.5–3.0 $\frac{\text{g}}{\text{L}}$ ), and it was demonstrated that the coating thickness enhanced with the DA concentration increment. Specifically, using the 0.5 $\frac{\text{g}}{\text{L}}\;$ DA solution resulted in a thin coating layer formation which was not readily visible, while a PDA layer with higher thickness was produced employing DA solution with the concentration of 3.0 $\frac{\text{g}}{\text{L}}$ . Furthermore, the layer roughness increased when the concentration of DA was augmented, as observed in the cross-section images in Figure 2D.

## 4 Conclusion

According to the ability of PDA as a surface modifier, this nature-inspired material has been widely employed in TE applications. Notably, certain conditions and parameters should be considered for the PDA polymerization process, including coating time, temperature, pH of Tris-HCl, and initial DA concentration, to acquire beneficial results. Even though the mentioned parameters have been given fixed values (24 h, room temperature, pH 8.5, and 2 $\frac{\text{mg}}{\text{ml}}$ ) in most investigations, some researchers think outside the box and change the factors to regulate the coating layer’s features.

There is still a long way to go regarding translating these innovative coated biomaterials into the clinic; consequently, successfully managing parameters together for furnishing the modified scaffolds with the desired properties has become a prominent goal in the preclinical experiments.
